# Examining the Acceptance and Use of AI-Based Assistive Technology Among University Students with Visual Disability: The Moderating Role of Physical Self-Esteem

**DOI:** 10.3390/bioengineering12101095

**Published:** 2025-10-11

**Authors:** Sameer M. Alnajdi, Mostafa A. Salem, Ibrahim A. Elshaer

**Affiliations:** 1 Education Technology Department, Faculty of Education and Arts, University of Tabuk, Tabuk 71491, Saudi Arabia; salnajdi@ut.edu.sa; 2 King Salman Center for Disability Research, Riyadh 11614, Saudi Arabia; 3 Deanship of Development and Quality Assurance, King Faisal University, Al-Ahsa 31982, Saudi Arabia; 4 Management Department, School of Business, King Faisal University, Al-Ahsa 31982, Saudi Arabia

**Keywords:** AI assistive technologies, visual impairment, UTAUT, physical self-esteem, technology adoption, higher education

## Abstract

AI-based assistive technologies (AIATs) are increasingly recognised as essential tools to enhance accessibility, independence, and inclusion for visually impaired students in higher education. However, limited evidence exists regarding the determinants of their acceptance and use, particularly in terms of psychosocial factors. This study aimed to extend the Unified Theory of Acceptance and Use of Technology (UTAUT) by incorporating physical self-esteem (PSE) as a moderator and behavioural intention (BI) as a mediator within a single model. Data were collected through a validated questionnaire administered to 395 visually impaired undergraduates across five Saudi universities. Constructs included effort expectancy (EE), performance expectancy (PE), facilitating conditions (FCs), social influence (SI), BI, and PSE. Partial Least Squares Structural Equation Modelling (PLS-SEM) was used for analysis. Results showed that PE and SI significantly predicted both BI and adoption, while EE strongly predicted BI but not AIAT adoption; FC had no significant influence on either outcome. BI positively affected AIAT adoption and mediated the effects of PE, EE, and SI, but not FC. Moderation analysis indicated that PSE strengthened the influence of PE, EE, and SI on BI and adoption. These findings underscore the significance of psychological factors, particularly self-esteem, in promoting the adoption of AIAT and offer guidance for developing inclusive educational strategies.

## 1. Introduction

Artificial intelligence-powered assistive technologies (AIATs) have become essential tools for supporting higher education students with disabilities in general, and those with visual impairments in particular [1]. Likewise, the adaptive solutions include text-to-speech applications, voice command systems, and intelligent navigation tools [2]. By enabling greater independence in education, AIATs not only promote academic accessibility but may also strengthen the role of physical self-esteem in shaping students’ adoption and use of such technologies [3].

Additionally, AIATs represent a transformative revolution in supporting individuals with visual impairments by bridging the gap between the digital and physical worlds, offering tools that enhance independence and interaction with the surrounding environment [4,5]. Moreover, in contrast to traditional assistive technologies, AIATs operate intelligent systems that continuously learn and adapt to users’ needs, offering advanced capabilities that significantly surpass those of conventional tools [6].

Beyond assistive capabilities, AIATs employ adaptive learning algorithms to analyse user interactions and contextual data, continuously optimising performance and tailoring experiences to individual needs [7]. Additionally, these systems refine functionality by learning from user behaviour and environmental cues, enabling applications such as real-time navigation for the visually impaired, smart home control, predictive text assistance, and integration with robotic systems for enhanced mobility and self-care [8].

Furthermore, through this dynamic adaptability, AIATs evolve from static tools into intelligent, personalised ecosystems that address both cognitive and physical challenges. However, concerns regarding affordability, algorithmic transparency, data privacy, and ethical governance underscore the need for ongoing human oversight to ensure the equitable and responsible implementation of these systems [8,9].

Despite advances, students with visual impairments still face barriers to adopting assistive technologies, including technological limitations, lack of institutional support, and psychological factors such as low self-esteem and the mediating role of physical self-esteem [10,11,12]. In the context of Saudi Arabia’s higher education, despite recent initiatives aimed at expanding inclusive digital infrastructure, empirical evidence on the determinants of AIAT adoption remains lacking [13,14,15]. Notably, there is a limited understanding of psychosocial factors—such as the mediating role of physical self-esteem—that influence the acceptance and use of these technologies.

Furthermore, UTAUT provides a robust framework for technology adoption. In addition, the four concepts of UTAUT—performance expectancy (PE) [16], effort expectancy (EE) [17], social influence (SI) [18], and facilitating conditions (FCs) [13]—have been widely validated in various educational settings. However, their role in explaining visually impaired students’ acceptance of AIAT adoption, and the extent to which behavioural intention (BI) mediates these relationships, remains insufficiently explored. Furthermore, few studies have investigated how individual psychological resources, such as physical self-esteem, moderate these relationships in the context of special needs education, particularly in higher education.

Accordingly, this study addresses these gaps by examining the acceptance and use of AIAT among visually impaired undergraduate students in Saudi Arabia, applying the UTAUT framework with the inclusion of mediating and moderating variables. The following research questions guide the study:

- ***RQ1:** How do PE, EE, SI, and FCs affect visually impaired students’ BI to adopt AIAT?*
- ***RQ2:** Do these constructions directly predict AIAT adoption?*
- ***RQ3:** Does BI mediate the relationship between UTAUT constructs and adoption of AIAT?*
- ***RQ4:** Does Physical self-esteem moderate the relationships between UTAUT constructs, BI, and AIAT adoption?*

By addressing these questions, the current study makes a significant contribution to both theoretical and applied research. On the theoretical side, the study extends the scope of the UTAUT model by incorporating physical self-esteem as a moderating variable in the context of assistive technologies. On the practical side, the study provides empirical evidence to guide policymakers, educators, and technology developers in formulating inclusive strategies for the effective adoption of AI technologies in higher education.

## 2. Literature Review

### 2.1. Self-Esteem and AI Assistive Technologies (AIATs) for Students with Visual Impairments

Higher levels of disability acceptance are often associated with stronger self-esteem among students with disabilities, particularly those with visual impairments [19,20]. Moreover, social and physical factors also play a significant role in shaping self-esteem, as positive peer relationships enhance mental health and foster greater self-worth [21,22]. Notably, recent studies such as [23,24,25,26] have further highlighted a positive association between body image—an individual’s perception of their own body—and self-esteem, particularly during the critical developmental stage of adolescence. Conversely, low levels of disability have consistently been linked to low self-esteem, a pattern frequently observed across different categories of disability [27,28,29].

Artificial intelligence (AI) has emerged as a transformative force in education, with profound implications for special needs contexts. Recent studies [30,31,32] have demonstrated how AI-powered assistive technologies (AIATs) have revolutionised digital learning by offering adaptive and interactive experiences specifically designed for students with disabilities, particularly those with visual impairments. Other research [13,33,34] shows that tools not only foster inclusivity but also enhance engagement and participation in the learning process.

AIATs—including applications such as advanced voice recognition systems, AI-powered writing assistants, and smartphone-based solutions like Siri, Pocket Vision, and Seeing AI—provide comprehensive support that helps individuals with disabilities overcome daily challenges [35,36,37]. Almufareh et al. [5] noted that these technologies promote independence, facilitate access to educational resources, and support social inclusion. Similarly, Venkatesh et al. [38] highlighted how AI and big data enhance the adaptability of assistive tools, thereby contributing to improved self-esteem among students with visual impairments.

In higher education, AIATs enable visually impaired students to access academic resources independently, participate in inclusive learning communities, and navigate technological barriers. Despite these benefits, most existing studies have concentrated on classifying AIATs, with relatively few examining the factors that influence their adoption. Moreover, research explicitly linking the use of AIATs to self-esteem outcomes remains limited. In the Saudi Arabian context, in particular, there is a notable gap in empirical studies investigating the impact of AIAT adoption on self-esteem among visually impaired students, especially when analysed within established theoretical frameworks, such as the UTAUT.

### 2.2. Technology Acceptance, Self-Esteem, and Research Hypotheses

The UTAUT provides a robust framework for analysing technology adoption. Widely applied in educational contexts, the UTAUT emphasises four key constructs—performance expectancy (PE), effort expectancy (EE), social influence (SI), and facilitating conditions (FCs)—as primary predictors of behavioural intention (BI) [39,40]. Likewise, the study extends the UTAUT framework by incorporating physical self-esteem as a moderating factor, acknowledging its potential to strengthen or weaken the influence of the core constructs on BI among visually impaired students [41,42].

In addition, PE describes the level to which people believe that using AIATs will improve their performance [38]. Within the context of AI-powered assistive technologies (AIATs), PE reflects the belief that tools such as screen readers and AI voice navigation systems can significantly improve learning outcomes, independence, and access to educational resources. Moreover, empirical evidence confirms that higher PE increases both intention and likelihood of AIAT adoption [43,44]. Therefore, the following hypotheses are assumed:

**H1:**  *PE is positively correlated with BI.*

**H2:**  *PE has a positive correlation with AIAT adoption.*

Furthermore, EE denotes the perceived ease associated with using a AIATs [38]. For visually impaired students, this encompasses the simplicity and intuitiveness of interacting with AIATs, including mobile applications, text-to-speech systems, and AI-powered writing assistants [44]. Similarly, technologies perceived as easier to use are more likely to be adopted and integrated into daily routines, with prior research confirming that EE contributes to higher user satisfaction and sustained adoption [45,46]. Accordingly, the following hypotheses are hypothesised:

**H3:**  *EE is positively correlated with BI.*

**H4:**  *EE positively correlated with AIAT adoption.*

Additionally, SI reflects the extent to which an individual perceives that significant others (e.g., peers, family, educators) believe they should use AIATs [47]. For visually impaired students, encouragement from these referent groups can reduce perceived stigma and increase confidence in adopting AIATs [13]. Likewise, research indicates that peer support and professional recommendations are significant factors shaping AIAT-related decisions [47,48]. Therefore, the following hypotheses are hypothesised:

**H5:**  *SI is positively correlated with BI.*

**H6:**  *SI is positively correlated with AIAT adoption.*

Furthermore, FCs refer to people’s belief that organisational and technical infrastructure exist to support the use of AIATs [49]. This includes access to compatible devices, reliable internet, adequate training, and timely technical support [50]. Notably, the presence of such conditions is a critical enabler of successful AIAT adoption [38,51]. Consequently, the following hypotheses are assumed:

**H7:**  *FCs are positively correlated with BI.*

**H8:**  *FCs are positively correlated with AIAT adoption.*

Additionally, BI is a well-established proximal predictor of AIAT adoption [52]. In this context, BI reflects a student’s conscious plan and willingness to utilise AIATs in their academic and daily activities [38]. Also, research consistently identifies BI as a strong direct determinant of use behaviour [50]. Furthermore, BI often serves as a mediating mechanism, linking UTAUT constructs to ultimate adoption outcomes [41,42]. As a result, the following hypotheses are proposed:

**H9:**  *BI is positively correlated with AIAT adoption.*

**H10:**  *BI mediates the relationship between PE and AIAT adoption.*

**H11:**  *BI mediates the relationship between EE and AIAT adoption.*

**H12:**  *BI mediates the relationship between SI and AIAT adoption.*

**H13:**  *BI mediates the relationship between FCs and AIAT adoption.*

Moreover, physical self-esteem refers to an individual’s subjective appraisal of their own physical competence and confidence in managing bodily appearance and daily tasks [52,53]. For visually impaired students, higher self-esteem may potentiate the positive influence of PE, EE, and SI on BI, whereas lower self-esteem may attenuate these relationships. Notably, incorporating this construct into the UTAUT framework adds a critical psychosocial dimension to understanding the drivers of AIAT adoption. Accordingly, the following moderation hypotheses are proposed:

**H14:**  *Physical self-esteem moderates the relationship between PE and BI.*

**H15:**  *Physical self-esteem moderates the relationship between EE and BI.*

**H16:**  *Physical self-esteem moderates the relationship between SI and BI.*

**H17:**  *Physical self-esteem moderates the relationship between PE and AIAT adoption.*

**H18:**  *Physical self-esteem moderates the relationship between EE and AIAT adoption.*

**H19:**  *Physical self-esteem moderates the relationship between SI and AIAT adoption.*

## 3. Methods

### 3.1. Instrument Development

The paper depends on a previously tested and validated scale to obtain data from visually impaired university students. The questionnaire was designed in five main parts. The first page contains the introductory paragraph, which provides a concise overview of the study objectives and includes a consent statement to secure respondents’ agreement to participate in this study. The first part was designed to collect demographic data, including respondents’ gender and year of study. The second section assessed the respondents’ physical self-esteem (moderator variables) based on the physical self-concept part of the “Physical Self-Description Questionnaire” (PSDQ) [54,55]. The PSDQ provided a comprehensive evaluation of students’ positive feelings about their physical self) [54,56]. Students were asked to evaluate this scale on a five-point Likert measure. The third part captured variables measuring the independent variables (performance expectancy—PE, effort expectancy—EE, and social influence—SI) and facilitating conditions—FCs. These dimensions were measured on a five-point Likert scale and were derived from previous studies [56,57], formerly coined as the “Unified Theory of Acceptance and Use of Technology” (UTAUT) framework [38,58]. The fourth part evaluated behavioural intention (BI) and AIAT adoption. Variables in this part were also derived from earlier validated scales [56,57], employing a five-point Likert scale (all the study measures are shown in Appendix A). To assess the adequacy of the employed measures, the designed questionnaire was first evaluated by 10 professors, who assessed the clarity, relevance, and appropriateness of the variables (questions). Following this phase, a pilot test was implemented on 10 university students (visually impaired) at King Faisal University (KFU). Comments from the two phases designated that the questions are easily understandable, necessitating only very minor linguistic changes. Together, these two phases offered a strong indication that the scale has adequate face and content validity.

To ensure full accessibility for visually impaired participants, the survey was designed and administered in multiple accessible formats. The online version of the questionnaire was developed in accordance with the Web Content Accessibility Guidelines (WCAG 2.1) and is fully compatible with standard screen-reader software, such as (JAWS (version 2024) and NVDA (version 2023.3.2)). Participants could navigate the survey independently using keyboard shortcuts and voice commands. Additionally, respondents who preferred oral assistance were supported by trained enumerators who read each question and response option verbatim, without paraphrasing or elaboration, to preserve standardisation. All enumerators received prior training and followed a structured script to ensure procedural consistency across all sessions. These accommodations ensured equitable participation and reliable data collection for all respondents, regardless of their level of visual ability.

### 3.2. Study Population and Sampling Calculation

According to the 2024 Saudi General Population and Housing Census, disabilities in KSA residents include impairments in hearing, mobility, cognition, self-care, communication, and vision. Out of the total KSA residents of 36 million, about 1.8% (around 648,000 residents) are alive with a form of disability. Notably, higher education students comprised a substantial proportion of this group, accounting for approximately 58% (n = 37,584), which reflected the growing number of students with disabilities in KSA universities. The report noted that the majority of university students with disabilities are enrolled in five major governmental universities: King Abdulaziz University (KAU) (1569 students), King Saud University (KSU) (663), Taibah University (TU) (523), Umm Al-Qura University (UQU) (381), and King Faisal University (KFU) (330). To serve the purpose of this study, only university students with visual impairment were involved, while excluding students with other disability patterns.

To determine the required sample size for the study, a power analysis was conducted using G*Power (version 3.1). The programme F-test family option was selected with the “Linear multiple regression: Fixed model, deviation from zero” choice. The output revealed that, to perceive a medium effect (f^2^ = 0.15) with six predictors (three independent variables, one mediator, and one moderator), a sample of 89 responses was required to achieve a power of 0.95 with a significance level of 0.05.

A convenience sampling approach was conducted to obtain the required sample size. To safeguard ethical and efficient data collection processes, 45 enumerators were recruited and trained to have enough research ethics (i.e., how to obtain informed consent and how to secure confidentiality and sensitivity when dealing with respondents). The training programme also introduced the study’s main objectives and offered methods for dealing with respondent’s concerns. Of the 950 distributed forms, 395 were fully completed and deemed valid, resulting in a 41.5% response rate.

To ensure equitable participation for visually impaired respondents, 45 trained enumerators administered questionnaires under strict ethical and procedural guidelines. Additionally, enumerators were trained in accessibility support, including the use of screen reader-compatible digital forms (JAWS and NVDA) and Braille-printed versions for participants who preferred tactile access. Regarding respondents requesting verbal assistance, enumerators read items aloud using a standardised script approved during pilot testing to prevent leading questions, unintentional cues, or bias. Participants completed responses independently whenever possible; no proxy responses were accepted. To protect privacy, all verbal assistance sessions were conducted individually in controlled settings, ensuring that respondents’ choices remained confidential.

Furthermore, fieldwork logs were maintained to record participation rates, refusals, and technical issues encountered during administration. Likewise, 950 distributed questionnaires yielded 395 valid responses, while nonresponses (n = 555) were primarily due to incomplete forms, withdrawal during the consent process, or difficulties with digital access. A nonresponse analysis revealed no statistically significant demographic differences between early and late respondents, suggesting limited nonresponse bias.

The demographic profile (as seen in Table 1) of the visually impaired university students signalled significant insights into the diversity and inclusivity of students with disabilities.

Out of the 395 replies, female students represented 54.2% (n = 214), while males accounted for 45.8% (n = 181). The balanced distribution of gender signalled that opportunities for visually impaired university students are not highly skewed by gender type, signifying progress in impartial access to higher education for both females and males with disabilities. The demographic statistics also demonstrated that the highest number of respondents are students at KFU (26%), KSU (22%), UOQ University (18%), TU (18%), and KAU (16%), respectively. Regarding academic discipline, the largest proportion of university students is registered in social sciences (38%), humanities (35.5%), and business administration (22.5%), while only 4% are enrolled in the applied sciences discipline accordingly. This distribution signalled that visually impaired university students may prefer study disciplines that are less reliant on visually based learning facilities such as laboratories and technical diagrams. The participants were divided into three levels, with 48% falling between 20 and 25 years old, 29% being below 20 years old, and 23% being above 25 years old. This age classification signalled that the majority of visually impaired students are aged in the conventional undergraduate age level (20–25 years). Nearly 25% of students over 25 years suggested that some visually impaired university students may enter education later, possibly due to delays in enrolment, rehabilitation requirements, or preparation prior to the transition to university. To better articulate the results of this study, respondents were asked to mention their prior experience and level of involvement with AIAT. The answers showed that although every respondent had some exposure to AIAT usage, the level of usage varied significantly. Approximately 33% described their exposure as occasional, used only when needed. A further 42% stated moderate usage, incorporating AIAT 2–4 times per week. The remaining 25% demonstrated that they are frequent and fully integrated participants, using AIAT daily.

### 3.3. Common Method Variance (CMV) Concerns

Concerns of CMV frequently occur in social science research papers as both independent and dependent measurement items are often self-reported by the same participants [59]. This issue may limit the explanatory and validity strength of the study model [60]. To address this potential bias, we followed the suggestions of [61] and employed statistical and procedural approaches. A procedural approach was developed to carefully design the questionnaire, ensuring balance across its parts, starting with the dependent items [61]. This structure aimed to minimise the order influence, dampen the foreseeable replies to patterns, and sustain an adequate overall questionnaire length. Statistically, CMV was tested using “Harman’s single-factor” method. The results indicated that the first extracted dimension represented only 41% of the total explained variance, which is below the 50% threshold. This outcome suggested that CMV was not a significant threat to the study’s results.

To further ensure data integrity and reduce the risk of common method variance (CMV), several procedural and statistical safeguards were implemented beyond the initial Harman’s single-factor assessment. A marker variable technique was used by including an unrelated construct (attitude toward campus safety) to test for CMV; the marker variable accounted for less than 2.5% of the shared variance with focal constructs, indicating no significant inflation of correlations.

Data quality controls were implemented throughout the collection process. Automated checks were applied to detect straight-lining, extreme outliers, and duplicate IP entries. Response durations falling below the pre-established 40% threshold of the median completion time were flagged and removed (n = 11). No duplicate submissions were retained. Assisted completions were independently monitored using enumerator logs to ensure loyalty to standardised reading procedures. Missing data analysis revealed that item-level missingness was below 2% across all variables, and Little’s MCAR test was non-significant (χ^2^ = 14.82, *p* = 0.26), supporting the assumption of random missingness. Given the low proportion, missing values were imputed using expectation–maximisation (EM) estimation prior to PLS-SEM analysis.

To minimise order effects and position bias, questionnaire sections were counterbalanced during pilot testing, and no statistically significant differences in mean construct scores were detected between split forms (*p* > 0.05). The final instrument retained the empirically validated order, starting with demographic items and ending with adoption-related questions, to ensure logical flow and respondent engagement. Collectively, these measures confirm that CMV, response bias, and order effects were not significant threats to data validity in this study.

### 3.4. Ethical Concerns

Taking into consideration the sensitive nature of our research (surveying university students with visual impairments), strict adherence to the ethical standards was ensured. Prior to the data collection process, a formal appeal was submitted to the “Institutional Review Board” (IRB) at KFU. Ethical approval was obtained under the number “KFU-REC-2025-APR-ETHICS3201” on 6 September 2024. This approval confirmed that the research paper was in accordance with the university guidelines and adhered to the ethical principles outlined in the Declaration of Helsinki [62]. To defend respondent’s rights and security, numerous protective methods were conducted. Contribution to the current study was completely voluntary, and no form of pressure was implemented. A written informed consent was attained from each participant after they were offered clear data concerning the study’s purposes. Additionally, respondents were clearly informed that they could withdraw from participation at any phase without justification. To safeguard the privacy and confidentiality of all respondents, all collected data were anonymised, thereby avoiding the identification of any distinct responses.

## 4. Data Analysis Methods and Results

To evaluate the justified hypotheses, “Partial Least Squares Structural Equation Modelling” (PLS-SEM) was used as the main analytical technique. PLS-SEM is a variance-oriented method, primarily suitable for exploratory and predictive-driven research due to its flexibility [63]. Contrasting the covariance-based SEM (CB-SEM), which classically requires a large sample size and strict adherence to multivariate normality, PLS-SEM can accommodate smaller sample sizes and does not depend on the normality assumption [64]. The data analysis was conducted employing the SmartPLS programme version 4 [65]. To ensure the robustness of the findings, a bootstrapping process with 5000 subsamples was performed using a reflective measurement procedure [66]. Following the recommendation from Hair et al. (2019) [67], the model assessment process was performed in two successive stages. The first stage was conducted to evaluate the measurement (outer) model to confirm its psychometric properties (adequate validity and reliability) (see Table 1). The second stage was conducted to assess the structural (inner) model (hypothesis testing) to determine whether the assumed research hypotheses can be approved or refuted.

### 4.1. Measurement Model Evaluation

**Figure 1:**
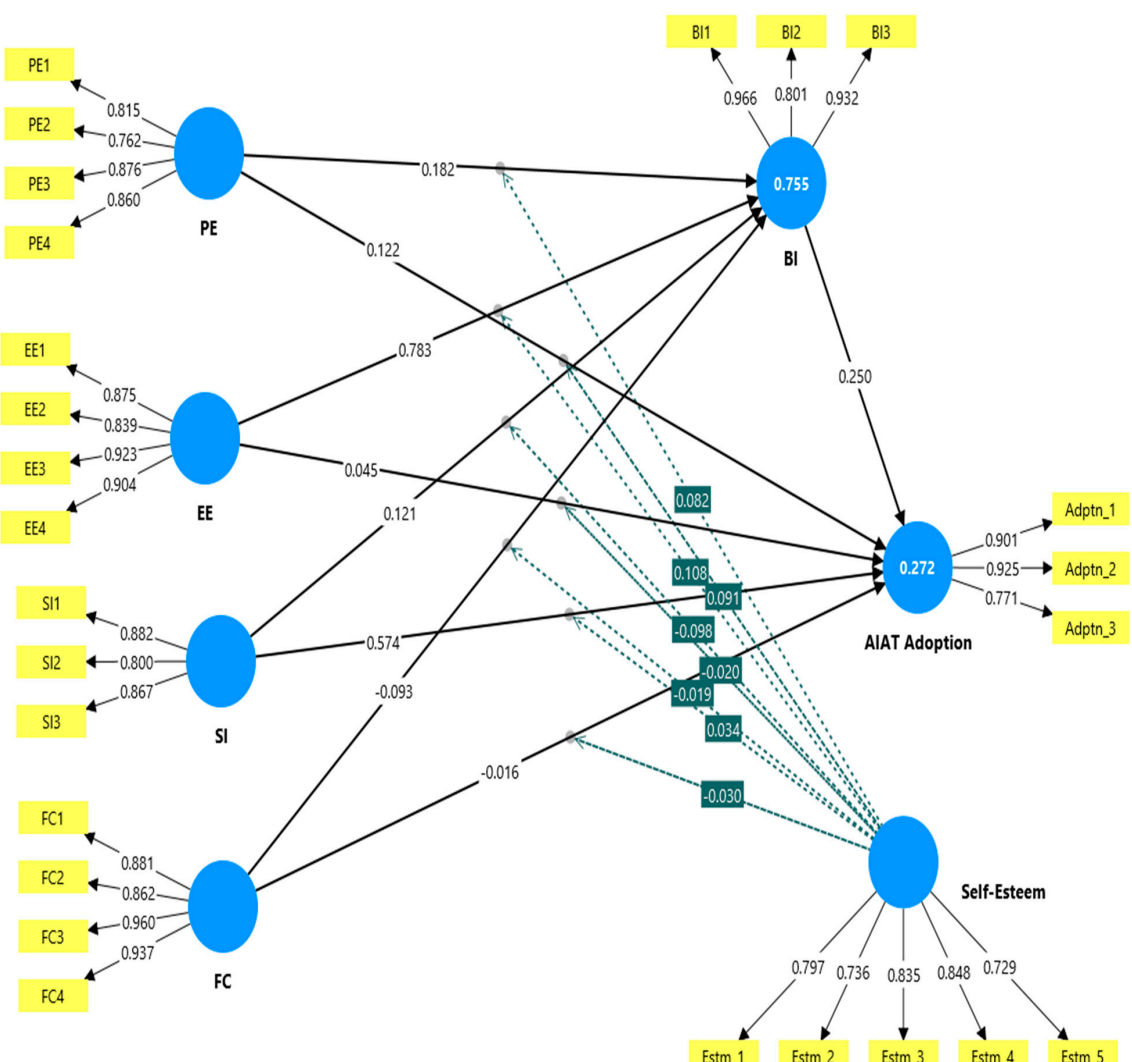
Study inner and outer models.

The assessment of the (outer) measurement model (as seen in Table 1 and Figure 1) was performed through an evaluation of four main psychometric criteria. First, the “standardised factor loadings” (SFLs) for all employed constructs were above the recommended threshold of 0.70, signalling a strong construct reliability. Second and third, both “Cronbach’s alpha” (α) and “composite reliability” (C.R) scores exceeded the value of 0.70, supporting an adequate level of reliability. Fourth, the “average variance extracted” (AVE) for each factor surpassed the minimum value of 0.50 [68]. Collectively, these indicators prove adequate internal consistency and good convergent validity. To further evaluate discriminant validity, three indicators were investigated. First, following the Fornell and Larcker (1981) [63] suggestions, the square root of each factor’s AVE (As seen in Table 2) was equated with its inter-factor correlations. As demonstrated in Table 3, the square root of AVE for each factor exceeded the corresponding intercorrelations, signifying adequate discriminant validity. Second, the “heterotrait–monotrait ratio”, as seen in Table 4 (HTMT) [64], was recognised as a more rigorous metric, and was evaluated. All HTMT scores were found to be below the critical cut-off of 0.90 (see Table 4), which further gives strong evidence of adequate discriminant validity. Third, a review of cross-loadings (Table 5) indicated that all items loaded adequately onto their intended factor, with no evidence of significant cross-loading, which further supports the adequate discriminant validity of the study construct.

### 4.2. Structural Inner Model Results (Hypotheses Testing)

Before evaluating the assumed research hypotheses, a set of goodness-of-fit (GoF) criteria was reviewed. These criteria include the “standardised root mean square residual” (SRMR), the “coefficient of determination” (R^2^), and “predictive relevance” (Q^2^) as suggested by Hair et al. (2019) [67]. The adequate thresholds are as follows: the SRMR score should be below 0.08, the R^2^ score should be 0.10 or higher, and the Q^2^ score should be greater than zero. The current tested model fulfilled all these criteria, supporting its adequate explanatory and predictive power. In more detail, the SRMR value is 0.071 and is below the suggested threshold. Additionally, all endogenous variables showed adequate and good predictive power: BI (R^2^ = 0.755; Q^2^ = 0.729) and AIAT adoption (R^2^ = 0.272; Q^2^ = 0.205). Furthermore, the multicollinearity concern was evaluated by calculating the “Variance Inflation Factor” (VIF). Consistent with the suggested guidelines of Hair et al. (2019) [67], VIF scores below 5 are considered acceptable; all items in our model met this metric, indicating the absence of multicollinearity issues (see Table 2). Although few items for facilitating conditions (FCs) and effort expectancy (EE) recorded VIF values slightly above 4.0, all remained below the conservative threshold of 5.0, suggesting acceptable levels of multicollinearity.

To complement the reported model fit indices, the model’s predictive power was further examined using PLSpredict [69]. The Q^2^ predict scores for both BI and AIAT were greater than the value of zero, signalling that the study model has predictive relevance. At the variable level, most variables showed lower root mean square error (RMSE) values for the PLS-SEM model compared to the LM benchmark, suggesting superior out-of-sample predictive ability.

As the measurement model was validated and the structural model showed adequate GoF, the analysis could proceed to test the research hypotheses. The bootstrapping outcomes for the structural path coefficients are shown in Table 5, revealing that performance expectancy (PE) of the AIAT framework was found to have a direct significant relationship with behavioural intention (BI) (β = 0.182, t = 3.539, *p* < 0.001) and AIAT adoption (β = 0.122, t = 1.997, *p* < 0.05) among visually impaired university students, supporting H1 and H2.

Similarly, effort expectancy (EE) was found to have a direct significant relationship with BI, showing a highly significant path coefficient (β = 0.783, t = 19.126, *p* < 0.001) and supporting H3, but failed to show a significant relationship with AIAT adoption (β = 0.045, t = 0.469, *p* = 0.639), rejecting H4. The PLS_SEM report also revealed that SI shows a significant relationship with the ability to increase BI (β = 0.121, t = 1.971, *p* < 0.05) and AIAT adoption (β = 0.574, t = 2.767, *p* < 0.01), supporting H5 and H6.

Interestingly, FC failed to demonstrate a significant positive relationship with BI (β = −0.093, t = 1.646, *p* = 0.095) and AIAT (β = −0.016, t = 0.325, *p* = 0.745) among visually impaired university students, resulting in the rejection of H7 and H8. Finally, BI was found to have a direct and positive relationship with AIAT adoption among visually impaired students in SA (r = 0.250, t = 2.073, *p* < 0.05), supporting H9 as seen in Table 6.

As seen in Table 5, the PLS-SEM report also showed mediating and moderating effects. BI successfully mediated the relationship between PE, EE, and SI on AIAT adoption, but failed to mediate the relationship between FC and AIAT adoption.

Notably, PSE was found to strengthen the significant effect of PE on both BI and AIAT adoption, as well as the effects of EE and SI on BI. However, it failed to moderate the other tested relationships, particularly those involving FC. Thus, this pattern reveals a partial moderating effect, suggesting that the relationship between self-esteem and other variables is not universal across all relationships within the model. Rather, PSE exerts its strongest effect within contexts where confidence, perceived ability, and social reinforcement play a central role in shaping behavioural intentions.

Furthermore, this nuanced result suggests that self-esteem enhances the motivational and perceptual determinants of AIAT adoption but may be less relevant when structural or institutional factors—such as infrastructure or resource support—are involved. Consequently, the moderating role of PSE should be interpreted as selective and context-dependent rather than general, reinforcing the importance of integrating psychological dimensions into technology acceptance models.

Regarding the moderating effects, the PLS-SEM revealed that PE shows a significant relationship with BI and AIAT adoption, which was stronger when visually impaired university students reported a high level of physical self-esteem.

Additionally, while the construct of PSE was rigorously examined as a pivotal moderating variable within the context of this study, it is also important to note that other psychological factors may exert a substantial influence on AIAT adoption, showing a significant relationship. Constructs such as resilience, technology self-efficacy, and self-autonomy could interact with the core UTAUT determinants to shape behavioural intentions and actual usage more comprehensively [70].

Moreover, integrating these variables into an expanded theoretical model may offer a more nuanced and holistic understanding of the psychological antecedents underlying technology adoption among individuals with visual impairments.

As a result, future studies should thus move beyond self-esteem alone to capture the interplay between motivational, cognitive, and emotional components of user psychology, thereby enriching the explanatory power of AIAT adoption frameworks.

Similarly, the results show a significant relationship of EE, BI, and SI with AIAT adoption, which was stronger when visually impaired university students reported a high level of physical self-esteem. However, physical self-esteem failed to moderate the other tested relationships, as seen in Table 5.

## 5. Discussion

The current study examined the adoption and usage of AI-based assistive technology (AIAT) among visually impaired university students in KSA, highlighting the moderating effects of physical self-esteem. By integrating the “Unified Theory of Acceptance and Use of Technology” (UTAUT) with self-perception theory, the results provided a deeper understanding of how technological and psychological dimensions can collectively shape technology adoption and usage in an inclusive educational environment.

The study results revealed that performance expectancy (PE) has a significant positive direct effect on both behavioural intention and actual AIAT usage among visually impaired university students in KSA. This result indicated that expectations of academic success through AIAT promote both BI and AIAT usage. This pattern aligns with prior evidence in the educational context, where PE has been reported to have a significant impact on students’ intentions to adopt AI-driven learning applications [71,72]. Similarly, Alshammari [73] argued that PE can predict the intention to use mobile applications when functionality and accessibility were recognised for visually impaired people. These results extended the applicability of the UTAUT framework—even among individuals with unique accessibility needs (i.e., visually impaired students).

Furthermore, the PLS-SEM results revealed that effort expectancy (EE) showed a high and significant impact on behavioural intention (BI). This suggests that visually impaired university students are more likely to intend to use AIAT when they recognise such AI tools as easy to operate and learn. This result is consistent with previous evidence that suggests when AIATs are perceived as accessible and user-friendly, university students with disabilities exhibit high usage intentions [74,75]. Though while EE significantly impacted BI, it did not show a direct impact on AIAT adoption. This suggests that the effectiveness of AIAT alone cannot be directly translated into actual sustained usage of AIAT. One probable reason is that preliminary intentions overemphasising perceived ease of use may encounter problems in real-life use, such as technical restrictions, accessibility concerns, or insufficient university support [73,76].

The results also showed that social influence (SI) can significantly predict both behavioural intention (BI) and AIAT adoption. These results suggest that support and inspiration from family, peers, and faculty members can play a key role in determining not only the intention but also the actual adoption of AIAT among visually impaired students in SA. These results are consistent with the assumption that SI is perceived as a critical element that can impact the adoption of technology [38], especially in SA, which is described as a collectivist society, where social influence and peer recommendations strongly impact decision-making [39]. Previous evidence has also demonstrated that students with disabilities rely heavily on peer and university support when deciding to be involved in new AI applications [77,78]. In contrast, facilitating conditions (FCs) had no significant impact on the adoption of both BI and AIAT. These results disagree with the UTAUT assumptions, which consider FCs as a predictor of actual usage [49]. A possible reason for these results might be related to the unique context of the study sample (visually impaired university students in SA). Even if a technical support system and AI infrastructure existed, university students may not recognise these facilities as sufficient enough for the adoption of AIAT. These outcomes are aligned with [79], who argued that the presence of AI infrastructure does not always guarantee useful adoption unless it is customised to fit learners’ accessibility requirements and accompanied by an adequate training programme and continuing support. Finally, the results of the current study model indicated that BI had a significant and positive direct impact on AIAT adoption. This aligns with prior evidence in general and disability-specific settings, where BI has been frequently employed as a strong predictor of technology adoption [74,80]. For visually impaired university students, this suggests that once a strong BI is developed—driven by PE, EE, and SI—it can efficiently be translated into adoption action.

The analysis of the mediation effects, as reported by the PLS-SEM programme, demonstrated that BI can successfully mediate the impact of PE, EE, and SI on AIAT adoption, but it was unsuccessful in mediating the influence of FCs on AIAT adoption.

The non-significant mediation effect of facilitating conditions (FCs) within the Unified Theory of Acceptance and Use of Technology (UTAUT) framework reflects underlying systemic barriers in how visually impaired students experience AI-driven assistive technology (AIAT) infrastructure. Despite the availability of AIATs in higher education institutions, these technologies are often inadequately integrated into learning management systems and broader instructional practices, consistent with the findings of [81,82].

Moreover, insufficient training among educators and technical staff in implementing AIATs—combined with the absence of accessible, AI-enhanced learning materials—undermines students’ confidence in the reliability and consistency of these systems. Consequently, the perceived facilitating conditions may not align with students’ actual accessibility experiences, resulting in reduced behavioural intention and lower adoption rates, consistent with the findings of [83,84].

This outcome likely stems from a persistent gap between institutional policies promoting digital inclusivity and the realities of classroom implementation. Such a disconnect creates an environment in which AIATs, though nominally available, remain underutilised or inaccessible in practice, thereby limiting their effectiveness. This contextual interpretation clarifies why FCs did not significantly predict adoption in the current study, despite their theoretically expected role within the UTAUT framework, consistent with the findings of [85].

These findings highlighted the significant role of BI as a psychological factor that can bridge the gap between beliefs and the actual adoption of AIAT. Similar evidence has been presented in previous studies that investigated the UTAUT framework, where BI consistently played a strong mediator role in technology usage, particularly in higher education and AIAT environments [74,77]. The non-significant mediation effects of BI in the relationship between FCs and AIAT suggest that even if support systems and infrastructure exist, they may not be directly translated into adoption unless university students possess strong BI. This result aligns with the findings of Chen et al. [86], who highlighted that limited accessibility, a lack of student-customised training programmes, and weak university support can undermine the recognised usefulness of FCs for university students with disabilities.

Regarding the moderating impacts, the findings revealed that students’ physical self-esteem can strengthen the impact of PE on both BI and AIAT adoption, suggesting that university students with higher self-confidence in their physical structure are more likely to perceive AIAT as valuable and then adopt it. This outcome aligns with previous evidence suggesting that a higher level of students’ self-esteem can enhance self-confidence in employing assistive technologies [86]. It is also aligned with self-determination theory, which suggests that a high self-concept enhances internal motivation to utilise supportive AI tools [87]. Likewise, the moderating effects of physical self-esteem were observed in the impact of EE on BI and between SI and AIAT adoption. This indicated that university students with a high level of physical self-esteem are more confident in their ability to manage technological demands (low effort expectancy) and are more responsive to social support, resulting in a higher level of adoption. Prior research on AI adoption among university students with disabilities suggests that self-esteem or self-efficacy may maximise resilience to perceived limitations and strengthen the impact of external motivators [5,78,80].

Interestingly, the moderating effect of PSE on the relationship between SI and BI was found to be significant but negative. This outcome suggests that higher levels of self-esteem may mitigate the impact of social approval or external expectations on students’ technology adoption decisions. Likewise, students with strong self-perceptions of capability and autonomy may rely less on peer or instructor influence when forming intentions to use AIATs. This finding aligns with self-determination theory, which posits that individuals with greater self-confidence tend to exhibit higher intrinsic motivation and reduced dependence on external validation [88,89].

In contrast, students with lower self-esteem may place greater weight on social cues, seeking affirmation and reassurance from their social environment before engaging with new technologies. Thus, the negative interaction observed here does not represent failed moderation but rather indicates a compensatory effect, where elevated self-esteem reduces susceptibility to social influence. This nuanced interpretation contributes to a more refined understanding of the psychological dynamics underlying assistive technology adoption among visually impaired students.

Nevertheless, physical self-esteem does not successfully moderate the other tested paths in the model. One possible reason is that self-esteem, while powerful in determining confidence and responsiveness to social cues, may not be sufficient to alter perceptions of FC.

## 6. Conclusions

This paper examined the intention and adoption of AIAT among visually impaired students in SA universities, employing the UTAUT framework with physical self-esteem acting as a moderator. The results suggested that PE, EE, and SI had a significant direct impact on BI, which in turn impacted the adoption of AIAT. Interestingly, while FCs are frequently hypothesised as critical factors in the adoption of AIAT, they were found to be unsuccessful in predicting either BI or AIAT adoption, highlighting the unique challenges visually impaired students might face when university support and infrastructure systems are unsatisfactory. Furthermore, the mediation analyses supported the key role of BI, which significantly mediated the influence of PE, EE, and SI on AIAT adoption, but failed to mediate the impact of FCs on AIAT adoption. This finding supports the importance of psychological factors compared to infrastructure support in this context. Additionally, the moderation analysis demonstrated that physical self-esteem strengthened the influences of PE, EE, and SI on BI and AIAT adoption, highlighting the key role of self-confidence in overcoming barriers to AIAT adoption. However, its impact was not universal, as it failed to moderate all the tested relationships.

The study provided some theoretical and practical implications. Theoretically, this study extended the UTAUT model by analytically testing the moderating key role of physical self-esteem, thus adding a psychological factor into the technology acceptance model. The study results challenged the classical assumption that FCs are constantly a direct antecedent of adoption, signifying that for some marginalised groups (i.e., disabled students), official support alone might not be sufficient without BI and self-confidence. Practically, policymakers in SA universities have to focus beyond just offering technological infrastructure systems (FCs) and emphasise creating students’ self-confidence and digital self-efficacy through customised training programmes and support systems to improve physical self-esteem and BI adoption of AIAT. For AI technology developers, AIAT applications should be customised to focus on ease of use (PE) and user-friendliness, as EE strongly impacts BI. Offering tailored and user-friendly systems can minimise perceived effort and promote the adoption of AIAT. For long-term sustainability, university managers should establish mentorship and colleague-support programmes to strengthen not only social influence but also students’ self-esteem, ensuring that AI adoption is not only created but can also be sustained over the long term.

## 7. Limitations and Future Research Opportunities

Similarly to other studies in the social sciences, the current research has several limitations that provide valuable opportunities for future investigation.

First, this study employed a cross-sectional research design, which, by its very nature, limits the ability to draw definitive causal inferences between the variables examined. Cross-sectional data capture responses at a single point in time, preventing the observation of how behavioural intentions and adoption patterns evolve as individuals gain experience with AI-driven assistive technologies. Given that AI adoption is a dynamic and iterative process influenced by ongoing exposure, training, and contextual adaptation, future studies should employ longitudinal or mixed-method research designs to better capture these temporal dynamics. Because causal claims cannot be made from the current study design, such approaches (longitudinal or mixed-methods research designs) would allow for the examination of causal pathways and developmental changes in students’ behavioural intentions and actual usage of AIATs over time, thereby offering stronger empirical validation of the UTAUT framework within accessibility contexts.

Second, the empirical data in this study were collected through self-reported surveys, a methodological approach that is inherently susceptible to distortions arising from Common Method Variance (CMV) and social desirability bias. Although several statistical and procedural remedies—such as ensuring respondent anonymity and applying Harman’s single-factor test—were employed to mitigate these risks, it is essential to acknowledge that such biases may persist to some extent, potentially influencing the observed relationships among constructs. Consequently, while the present results provide valuable insights into the behavioural intentions and adoption of AI-driven assistive technologies, the reliance on self-reported data may complicate the precision of causal interpretations. To enhance the robustness and validity of future research, scholars are encouraged to incorporate objective measures—such as digital usage logs, system analytics, or triangulated data sources—to complement self-reported responses. This would allow for a more accurate and nuanced understanding of AIAT adoption behaviours among visually impaired learners.

Third, in contrast to the anticipations derived from the UTAUT framework, facilitating conditions (FCs) did not exhibit a statistically significant influence on either behavioural intention (BI) or the adoption of AIAT. This finding is particularly noteworthy, as FCs are traditionally conceptualised as a critical determinant of technology acceptance, reflecting the availability of institutional support, infrastructure, and resources required for effective system use. The present results may stem from the distinctive context of the study population—visually impaired students in higher education—who often encounter institutional and infrastructural limitations that hinder the practical utilisation of AI-driven assistive technologies. Even when formal support systems exist, they may not be perceived as adequate, accessible, or responsive to students’ specific needs, thereby diminishing the predictive strength of FCs. These insights highlight the importance of incorporating more objective measures of AIAT engagement, such as system usage tracking or interaction data, in future studies. Such triangulated approaches would yield a more comprehensive understanding of how facilitating conditions and user experiences interact to shape technology adoption behaviours.

Fourth, although the current study examined physical self-esteem as a key moderating variable, other psychological constructs—such as resilience, technology self-efficacy, and self-autonomy—were not explored. Future studies should consider developing an expanded model that integrates a broader range of personal characteristics to understand the psychological antecedents of AIAT adoption better. Additionally, given the key role of gender in reshaping learning experiences and AIAT adoption for visually impaired students, future research should employ gender as a moderating variable by performing gender-stratified analyses. Prior research has already highlighted the importance of demographic factors in similar contexts (see Thakuret al. [90]; and Hudders & De Jans [91]). Therefore, future papers can offer a more nuanced understanding of how gender can influence the AIAT adoption in higher education.

Finally, this research primarily investigated AI-based assistive technologies in an educational context, overlooking other domains, such as the workplace and daily living, where these technologies may also play equally significant roles. Future studies could therefore explore how AIAT adoption influences overall quality of life (QoL) beyond academic performance, thereby broadening the understanding of AI’s role in enhancing inclusivity and accessibility across diverse contexts.

## Figures and Tables

**Table 1:** Statistics of respondents’ demographics.

Respondents’ Profile (N = 395)		No.	%
Gender	Females	214	54.2%
	Males	181	45.8%
University name	KAU	61	16%
	KSU	85	22%
	TU	69	18%
	UQU	72	18%
	KFU	103	26%
Academic discipline	Business administration	90	22.5%
	Humanities	140	35.5%
	Social sciences	150	38%
	Applied sciences	15	4%
Age	<20 years	115	29%
	20–25 years	190	48%
	>25 years	90	23%
Prior usage or adoption of AIAT	Occasional usage (for specific duties only)	130	33%
	Moderate usage (2–4 times weekly)	167	42%
	Frequent/integrated usage (almost daily use)	98	25%

**Table 2:** The psychometric properties of the study variables.

	FL	α	C.R.	AVE	VIF
(AIAT Adoption)		0.839	0.901	0.754	
(Adptn_1)	0.901				2.292
(Adptn_2)	0.925				2.943
(Adptn_3)	0.771				1.723
(Behavioural Intention)		0.884	0.929	0.814	
(BI1)	0.966				2.410
(BI2)	0.801				1.852
(BI3)	0.932				2.028
(Effort Expectancy)		0.908	0.936	0.784	
(EE1)	0.875				2.339
(EE2)	0.839				2.132
(EE3)	0.923				4.066
(EE4)	0.904				4.074
(Self-Esteem)		0.850	0.892	0.625	
(Estm_1)	0.797				2.603
(Estm_2)	0.736				1.812
(Estm_3)	0.835				1.090
(Estm_4)	0.848				1.124
(Estm_5)	0.729				2.358
(Facilitating Conditions)		0.908	0.936	0.784	
(FC1)	0.881				4.087
(FC2)	0.862				4.092
(FC3)	0.960				3.767
(FC4)	0.937				4.336
(Performance Expectancy)		0.851	0.898	0.688	
(PE1)	0.815				2.005
(PE2)	0.762				1.531
(PE3)	0.876				2.819
(PE4)	0.860				2.862
(Social Influence)		0.820	0.887	0.723	
(SI1)	0.882				1.581
(SI2)	0.800				2.135
(SI3)	0.867				2.589

**Table 3:** Fornell and Larcker metrics.

	AIAT Adoption	EE	FC	PE	SI	Self-Esteem
AIAT Adoption	0.868					
BI	0.432	0.902				
EE	0.378	0.829	0.886			
FC	−0.101	−0.216	−0.121	0.911		
PE	0.074	0.124	0.007	−0.065	0.829	
SI	0.385	0.357	0.374	−0.063	−0.220	0.850
Self-Esteem	0.309	0.275	0.299	−0.079	−0.356	0.731

**Table 4:** “Heterotrait–monotrait ratio” (HTMT) matrix.

	AIAT Adoption	EE	FC	PE	SI	Self-Esteem
AIAT Adoption						
BI	0.498					
EE	0.408	0.907				
FC	0.096	0.213	0.127			
PE	0.084	0.143	0.052	0.073		
SI	0.419	0.395	0.405	0.067	0.272	
Self-Esteem	0.351	0.316	0.338	0.083	0.409	0.123

**Table 5:** Loadings and factor cross loadings.

	AIAT Adoption	BI	EE	FC	PE	SI	Self-Esteem
Adptn_1	0.901	0.448	0.419	−0.132	0.078	0.394	0.334
Adptn_2	0.925	0.391	0.327	−0.105	0.058	0.329	0.254
Adptn_3	0.771	0.248	0.197	0.006	0.053	0.259	0.192
BI1	0.347	0.966	0.886	−0.192	0.096	0.310	0.236
BI2	0.499	0.801	0.502	−0.232	0.158	0.334	0.259
BI3	0.354	0.932	0.814	−0.172	0.094	0.331	0.257
EE1	0.445	0.781	0.875	−0.080	0.060	0.299	0.233
EE2	0.308	0.681	0.839	−0.089	−0.040	0.316	0.249
EE3	0.318	0.746	0.923	−0.154	−0.013	0.356	0.296
EE4	0.251	0.720	0.904	−0.107	0.007	0.357	0.285
Estm_1	0.283	0.244	0.243	−0.072	−0.488	0.694	0.797
Estm_2	0.201	0.215	0.240	−0.053	−0.170	0.761	0.736
Estm_3	0.238	0.152	0.168	−0.047	−0.179	0.817	0.835
Estm_4	0.263	0.193	0.248	−0.041	−0.168	0.822	0.848
Estm_5	0.223	0.264	0.269	−0.092	−0.337	0.604	0.729
FC1	−0.029	−0.102	−0.076	0.881	−0.021	−0.039	−0.051
FC2	−0.065	−0.138	−0.113	0.862	−0.003	−0.055	−0.068
FC3	−0.110	−0.182	−0.128	0.960	−0.036	−0.069	−0.085
FC4	−0.117	−0.276	−0.111	0.937	−0.119	−0.058	−0.074
PE1	0.071	0.063	−0.031	−0.028	0.815	−0.273	−0.388
PE2	0.095	0.119	0.023	−0.032	0.762	−0.128	−0.239
PE3	0.044	0.108	0.013	−0.080	0.876	−0.170	−0.284
PE4	0.021	0.107	0.004	−0.078	0.860	−0.190	−0.297
SI1	0.434	0.388	0.406	−0.064	−0.190	0.882	0.507
SI2	0.222	0.254	0.267	−0.048	−0.185	0.800	0.448
SI3	0.256	0.215	0.223	−0.043	−0.190	0.867	0.429

**Table 6:** Hypotheses evaluation.

Assumed Hypotheses	Path Coefficients	T Statistics	*p* Values	Evaluation
Hypothesis 1: PE -> BI	0.182	3.539	0.000	Approved
Hypothesis 2: PE -> AIAT Adoption	0.122	1.997	0.043	Approved
Hypothesis 4: EE -> BI	0.783	19.126	0.000	Approved
Hypothesis 4: EE -> AIAT Adoption	0.045	0.469	0.639	Rejected
Hypothesis 6: SI -> BI	0.121	1.971	0.043	Approved
Hypothesis 5: SI -> AIAT Adoption	0.574	2.767	0.006	Approved
Hypothesis 7: FC -> BI	−0.093	1.646	0.095	Rejected
Hypothesis 8: FC -> AIAT Adoption	−0.016	0.325	0.745	Rejected
Hypothesis 9: BI -> AIAT Adoption	0.250	2.073	0.038	Approved
Mediating effects				
PE -> BI -> AIAT Adoption	0.146	1.963	0.049	Approved
EE -> BI -> AIAT Adoption	0.196	2.089	0.037	Approved
SI -> BI -> AIAT Adoption	0.130	1.980	0.027	Approved
FC -> BI -> AIAT Adoption	−0.023	1.658	0.097	Rejected
Moderating effects				
Self-Esteem x PE -> BI	0.082	2.805	0.005	Approved
Self-Esteem x PE -> AIAT Adoption	0.091	2.035	0.042	Approved
Self-Esteem x EE -> BI	0.108	3.055	0.002	Approved
Self-Esteem x EE -> AIAT Adoption	−0.020	0.425	0.671	Rejected
Self-Esteem x SI -> AIAT Adoption	0.134	2.765	0.024	Approved
Self-Esteem x SI -> BI	−0.098	3.564	0.000	Rejected
Self-Esteem x FC -> BI	−0.019	0.701	0.483	Rejected
Self-Esteem x FC -> AIAT Adoption	−0.030	0.528	0.598	Rejected

## Data Availability

The data presented in this study are available on re-quest from the corresponding author due to its privacy.

## Appendix A. The Study Measures

	**Scale Variables and Items**
	Performance Expectancy (PE)
PE1	AIAT is a valuable tool for my academic pursuits
PE2	Utilizing AIAT improves the probability of attaining important objectives in your academic pursuits
PE3	AIAT enhances productivity in academic studies by expediting the completion of tasks and projects
PE4	Using AIAT can elevate my academic performance
	Effort Expectancy (EE)
EE1	I find it easy to learn how to use AIAT.
EE2	Communication with AIAT is transparent and easy to comprehend
EE3	AIAT is user-friendly and intuitive
EE4	I find it effortless to acquire expertise in using AIAT
	Social Influence (SI)
SI1	People play a crucial role in my life are of the opinion that I should utilize AIAT.
SI2	People shape my behavior recommend the utilization of AIAT
SI3	Those whose opinions I highly esteem suggest that I make use of AIAT
	Facilitating Conditions (FC)
FC1	I am adequately equipped with the necessary resources to make use of AIAT
FC2	I am proficient in utilizing AIAT due to acquired knowledge
FC3	AIAT is suitable for the technologies I utilize.
FC4	When facing difficulties with AIAT, it is possible to receive support and aid from external sources
	Behavioral Intention (BI)
BI1	I have decided to continue using AIAT in the times ahead
BI2	I am dedicated to utilizing AIAT as a tool for my studies
BI3	I aim to continue using AIAT on a frequent basis
	AIAT Adoption
Adptn_1	I intend to use the knowledge and skills I acquired from the AIAT in my educational activities.
Adptn_2	The knowledge and skills I acquired from AIAT will be useful to me in class.
Adptn_3	Using AIAT has helped to improve my academic performance
	Self-esteem
Estm_1	Physically. I am happy with myself,
Estm_2	Physically. I feel good about myself,
Estm_3	I feel good about who I am and what I can do physically.
Estm_4	I feel good about who I am physically.
Estm_5	I feel good about the way I look and what I can do physically
